# Synthesis of a tricyclic lactam via Beckmann rearrangement and ring-rearrangement metathesis as key steps

**DOI:** 10.3762/bjoc.11.163

**Published:** 2015-08-27

**Authors:** Sambasivarao Kotha, Ongolu Ravikumar, Jadab Majhi

**Affiliations:** 1 Department of Chemistry, Indian Institute of Technology-Bombay, Powai, Mumbai-400 076, India, Fax: 022-25767152

**Keywords:** allylation, Beckmann rearrangement, lactams, oximes, ring-rearrangement metathesis

## Abstract

A tricyclic lactam is reported in a four step synthesis sequence via Beckmann rearrangement and ring-rearrangement metathesis as key steps. Here, we used a simple starting material such as dicyclopentadiene.

## Introduction

**Figure 1:**
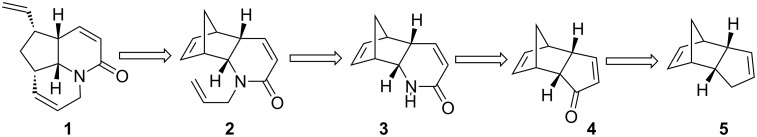
Retrosynthetic analysis of tricyclic amide **1**.

The Beckmann rearrangement (BR), a well-known protocol for the conversion of ketoxime to an amide in the presence of acid was discovered in 1886. This rearrangement involves the migration of a group anti to the leaving group on the nitrogen atom. The BR has widely been used in synthetic organic chemistry, for example, a large-scale production of Nylon-6 is based on the synthesis of ε-caprolactam from cyclohexanone oxime involving the BR. The activation energy for the BR is almost the same as that of the nucleophilic substitution at sp^2^ nitrogen. To synthesize various aza-arenes and cyclic imines, such as quinolines, aza-spiro compounds and dihydropyrroles, the intramolecular S_N_2-type reaction at the oxime nitrogen is useful [1–6]. Here, we plan to use the BR in combination with a ring-rearrangement metathesis (RRM) [7–24] to generate lactam derivative **1**. The RRM protocol involves a tandem process with several metathetic transformations such as ring-closing metathesis (RCM) and ring-opening metathesis (ROM). The RRM has emerged as a powerful tool in organic synthesis because of its ability to transform simple starting materials into complex targets involving an ingenious design. The retrosynthetic strategy to the target molecule **1** is shown in Figure 1. RRM of the tricyclic allylated compound **2** can deliver the target lactam **1**. The key synthon **2** can be derived by allylation of lactam **3**, which in turn can be prepared via BR starting with the known enone **4** [25–27], derived from dicyclopentadiene (**5**) [28–30].

## Results and Discussion

**Scheme 1:**
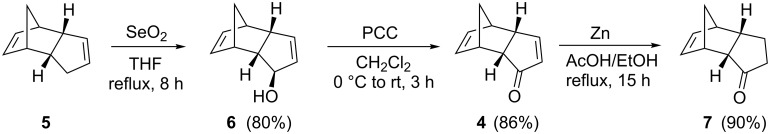
Synthesis of tricyclic ketone **4**.

To begin with, the oxidation of dicyclopentadiene (**5**) in the presence of SeO_2_ gave 1α-dicyclopentadienol (**6**), which on treatment with pyridinium chlorochromate (PCC) [31] delivered the known tricyclic enone **4**. Selective reduction of enone **4** with Zn in AcOH/EtOH under reflux conditions gave the saturated ketone **7** [32] (Scheme 1).

**Scheme 2:**
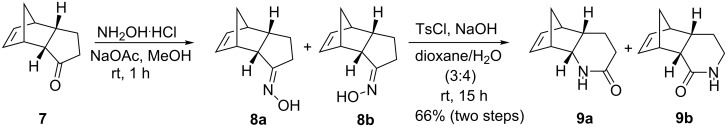
Beckmann rearrangement of oximes **8a** and **8b**.

Later, tricyclic ketone **7** was reacted with NH_2_OH·HCl in the presence of NaOAc in dry MeOH at rt to give a mixture of oximes **8a** and **8b** and this mixture was subjected to a BR under different reaction conditions, like (a) *p*-TsCl, rt, 15 h, CH_3_CN (b) *p-*TsCl, reflux, 15 h, CH_3_CN (c) PPA, reflux for 20 min. Surprisingly, in all these instances no rearrangement product was observed. Interestingly, when the mixture of oximes **8a** and **8b** was treated with TsCl in the presence of NaOH at rt lactams **9a** and **9b** were obtained in 66% combined yield for two steps (**9a**:**9b** = 2:1) (Scheme 2) but the products were inseparable by column chromatography. Next, we attempted to separate the mixture of these isomers (**9a** and **9b**) by selective crystallization using different solvent systems. Finally, one of the lactam derivative **9a** (δ = 3.86, dd, *J* = 5.8, 2.9 Hz, 1H) was isolated in pure form from ethanol in 20% yield over two steps.

**Scheme 3:**
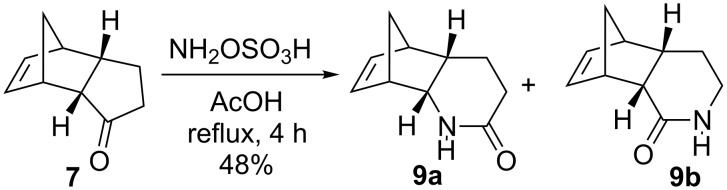
Beckmann rearrangement reaction in a single step.

Subsequently, we attempted to synthesize the desired lactam **9a** via Schmidt reaction or BR of the keto derivative **7** in a single step. In this regard, the tricyclic ketone **7** was treated under different reaction conditions. These include: (a) NaN_3_, heat 1 day in TFA (b) NaN_3_, FeCl_3_ in DCE at rt and reflux, 1 day and (c) TMSN_3_, FeCl_3_ in DCE, 1 day. Surprisingly, the desired lactam **9a** was not formed. Interestingly, when the tricyclic ketone **7** was treated with hydroxylamine-*O*-sulfonic acid (NH_2_OSO_3_H) in glacial AcOH under reflux conditions, the lactams **9a** and **9b** were obtained in 48% yield (**9a**:**9b** = 2:1) the ratio of oximes **9a** and **9b** was calculated based on ^1^H NMR spectral data (Scheme 3).

**Scheme 4:**
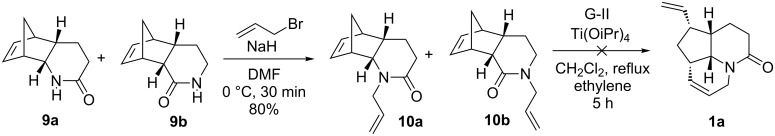
Synthesis of ring-rearrangement precursors.

Having prepared the lactams **9a** and **9b**, the allylation reaction was attempted with the lactam mixture in the presence of NaH/allyl bromide in dry DMF to generate allyl derivatives **10a** and **10b** in 84% yield. Later, without separation of allyl lactams **10a** and **10b**, RRM was attempted with the lactam mixture under different catalyst conditions. For example, reaction conditions such as: (a) G-I in dry CH_2_Cl_2_, under ethylene atmosphere at rt; (b) G-II in dry CH_2_Cl_2_, under ethylene atmosphere at rt and (c) G-I and G-II in dry toluene under ethylene atmosphere did not deliver the desired RRM product **1a** (Scheme 4).

**Scheme 5:**
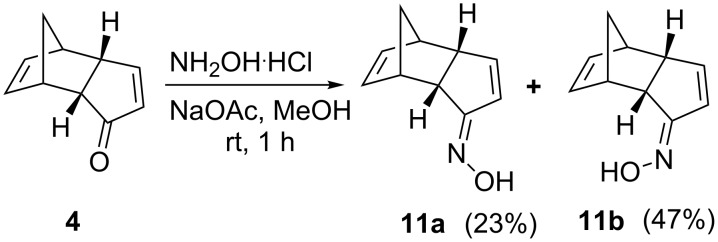
Synthesis of Beckmann rearrangement precursors.

Separation of the required isomer from the mixture of oximes **8a** and **8b** or the lactams **9a** and **9b** was not possible by column chromatography because of the same *R*_f_ value of the individual compounds. Finally, isolation of the required lactam **9a** from the mixture was accomplished by using crystallization. Since this method is cumbersome, an alternate method was attempted. To this end, we changed our synthetic route and tried to use the unsaturated ketone **4** and hoped for a different outcome during the BR. In this content, oximation of the enone **4** was carried out with NH_2_OH·HCl in the presence of NaOAc in dry MeOH. The stereoisomeric oximes, i.e., (*E*)-**11b** and (*Z*)-**11a** were separated by silica gel column chromatography to deliver 47% and 23% yields, respectively (Scheme 5).

**Scheme 6:**
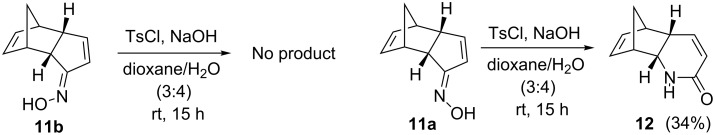
Beckmann rearrangement of oxime isomers **11a** and **11b**.

**Figure 2:**
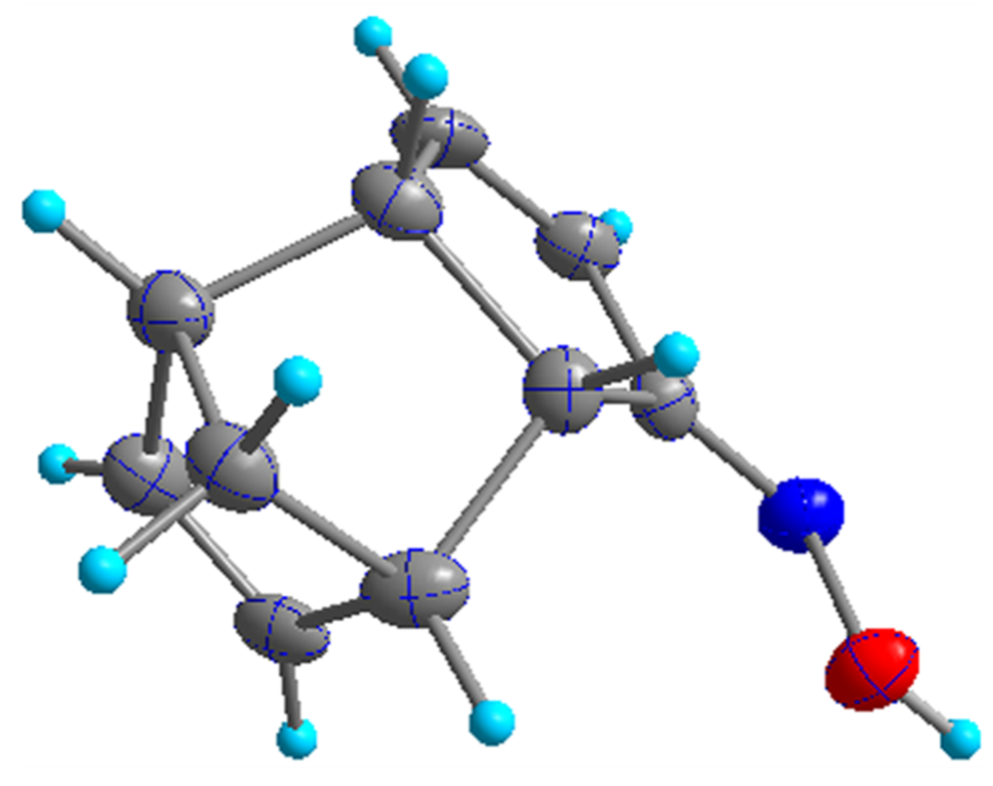
Molecular crystal structure of compound **11b**.

When the oxime **11a** was treated with TsCl in the presence of NaOH in dioxane/H_2_O (3:4 v/v) at rt lactam **12** was formed in 34% yield. However, the oxime **11b** did not give the rearranged product under the same reaction conditions, which clearly indicates that the oxime **11b** is unreactive towards BR (Scheme 6). The stereostructure of the oxime **11b** has been determined by single crystal X-ray diffraction data (Figure 2) [33].

**Scheme 7:**
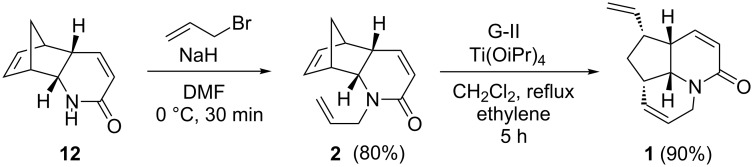
Synthesis of aza tricyclic compound **1** by RRM.

Allylation of lactam **12** in the presence of NaH/allyl bromide in dry DMF gave the allyl derivative **2** in 80% yield. Finally, the RRM of compound **2** was accomplished with G-II catalyst in dry CH_2_Cl_2_, under ethylene atmosphere at rt in the presence of Ti(OiPr)_4_ to deliver the tricyclic compound **1** in 90% yield (Scheme 7). Its structure has been established on the basis of ^1^H NMR and ^13^C NMR spectral data and further supported by HRMS data.

## Conclusion

In summary, we have demonstrated the RRM strategy with the norbornene derivative **2** to deliver the tricyclic compound **1** involving a short synthetic sequence. However, a similar compound **10a** did not deliver the RRM product. For the first time, we have demonstrated that BR in combination with RRM is a useful strategy to generate azacyclic compounds. Here we have used an inexpensive starting material such as dicyclopentadiene (**5**).

## Experimental

### Synthesis of compounds **9a** and **9b**

**Method 1:** Analogously as described in [4], a mixture of **7** (2 g, 13.51 mmol), hydroxylamine hydrochloride (1.41 g, 20.27 mmol), NaOAc (1.66 g, 20.27 mmol) and methanol (50 mL) was stirred at rt for 1 h. The residue after evaporation of the solvent was diluted with water and extracted with ether. Removal of ether furnished the crude oxime (2.4 g). *p*-Toluenesulfonyl chloride (6.15 g, 32.28 mmol) was added portion-wise over 15 min to a stirred solution of the crude oxime (2.4 g) and NaOH (2.97 g. 74.44 mmol) in 150 mL dioxane/water 3:4 at 5 °C. The mixture was stirred at rt for 15 h and dioxane was removed in vacuo. The residue was dissolved in CH_2_Cl_2_ and washed with brine. Removal of the solvent and column chromatography gave a mixture of amide isomers (**9a**, **9b**) (1.45 g, 66%). The amide mixture was crystalized in different solvents and finally one of isomer **9a** was isolated from ethanol 20%.

**Method 2:** A mixture of **7** (100 mg, 0.68 mmol) and hydroxylamine-O-sulfonic acid (113 mg, 1.0 mmol) in AcOH (5 mL) was heated at reflux conditions for 4 h under nitrogen. After completion of the reaction (TLC monitoring), the reaction mixture was basified with 3 N NaOH solution and the organic layer was extracted with CH_2_Cl_2_, washed with water, brine and dried by Na_2_SO_4_. The combined organic layer was concentrated under reduced pressure and column chromatography gave a mixture of amide isomers **9a** and **9b** (1.06 g, 48%). The amide mixture was crystalized in different solvents and finally isomer **9a** was isolated from ethanol. White solid **9a**; mp = 150–155 °C; yield 15%: *R*_f_ = 0.30 (EtOAc/pentane 1:1 v/v); IR (neat): 3195 (m), 3067 (w), 2938 (s), 2868 (m), 1674 (s), 1627 (m), 1452 (w), 1434 (w), 1410 (m), 1333 (m), 1252 (w), 1201 (m), 1031 (w), 783 (m), 541 (m) cm^−1^; ^1^H NMR (400 MHz, CDCl_3_) δ 6.31 (s, 1H), 6.22 (dd, *J* = 5.8, 3.0 Hz, 1H), 6.10 (dd, *J* = 5.8, 3.0 Hz, 1H), 3.86, (dd, *J* = 5.8, 2.9 Hz, 1H), 2.97 (s, 1H), 2.88 (s, 2H), 2.48–2.40 (m, 1H), 2.13–2.05 (m,1H), 1.94–1.87 (m, 1H), 1.56 (dt, *J* = 8.8, 1.8 Hz, 1H), 1.42 (d, *J* = 8.8 Hz, 1H), 1.23–1.13 (m, 1H) ppm; ^13^C NMR (100 Hz, CDCl_3_) δ 175.5, 137.6, 134.2, 54.8, 48.0, 47.8, 46.5, 39.49, 31.4, 23.2 ppm.

### Synthesis of compounds **11a** and **11b**

Analogously as described in [4], a mixture of **4** (9 g, 61.64 mmol), hydroxylamine hydrochloride (6.41 g, 92.34 mmol), NaOAc (7.58 g, 92.49 mmol) and methanol (225 mL) were stirred at rt for 1 h. The residue after evaporation of the solvent was diluted with water and extracted with diethyl ether. Removal of ether furnished the crude oxime which was purified by silica gel column chromatography by eluting appropriate mixture of ethyl acetate/petroleum ether to afford compounds **11a** (2.29 g, 23%) and **11b** (4.61 g, 47%) as colourless solids.

**11a**: *R*_f_ = 0.29 (EtOAc/pentane 2:8 v/v); IR (neat): 3325 (m), 3013 (m), 2400 (w), 1725 (w), 1337 (w), 1216 (m), 927 (m), 759 (s) cm^−1^; ^1^H NMR (500 MHz, CDCl_3_) δ 9.15 (s, 1H), 6.54 (dd, *J* = 5.8, 1.3 Hz, 1H), 6.38 (dd, *J* = 5.8, 2.5 Hz, 1H), 5.97 (dd, *J* = 5.6, 2.8 Hz, 1H), 5.77 (dd, *J* = 5.6, 2.9 Hz, 1H), 3.32 (m, 1H), 3.18 (dd, *J* = 10.7, 4.5 Hz, 1H), 3.16 (s, 1H), 2.28 (s, 1H), 2.90 (s, 1H), 1.61 (d, *J* = 8.3 Hz, 1H), 1.47 (d, *J* = 8.3 Hz, 1H) ppm; ^13^C NMR (125 Hz, CDCl_3_) δ 165.1, 149.1, 133.3, 133.1, 126.4, 51.0, 50.5, 46.1, 45.9, 44.1 ppm; HRMS (Q-Tof) *m*/*z*: [M + Na]^+^ calcd for C_10_H_11_NNaO, 184.0733; found, 184.0734.

**11b**: mp = 89–91 °C; *R*_f_ = 0.30 (EtOAc/petroleum ether 2:8 v/v); IR (neat): 3322 (m), 3020 (m), 2396 (w), 2125 (w), 1705 (m), 1217 (m), 926 (m), 759 (s) cm^−1^; ^1^H NMR (500 MHz, CDCl_3_) δ 8.71 (s, 1H), 6.30 (dd, *J* = 5.8, 2.5 Hz, 1H), 6.00 (dd, *J* = 5.7, 1.3 Hz, 1H), 5.90 (dd, *J* = 5.6, 3.0 Hz, 1H), 5.76 (dd, *J* = 5.6, 2.9 Hz, 1H), 3.43 (s, 1H), 3.35 (dd, *J* = 6.1, 4.2 Hz, 1H), 3.30 (m, 1H), 2.90 (s, 1H), 1.64 (d, *J* = 8.3 Hz, 1H), 1.50 (d, *J* = 8.3, 1H) ppm; ^13^C NMR (125 Hz, CDCl_3_) δ 168.1, 147.0, 133.1, 132.9, 131.1, 51.9, 50.8, 45.1, 45.0, 44.1 ppm; HRMS (Q-Tof) *m*/*z*: [M + Na]^+^ calcd for C_10_H_11_NNaO, 184.0733; found, 184.0737.

### Synthesis of compound **12**

Analogously as described in [4], *p*-toluenesulfonyl chloride (2.36 g, 12.42 mmol) was added portionwise over 15 min to a stirred solution of oxime **11a** (1.0 g, 6.21 mmol) and NaOH (1.24 g. 31.05 mmol) in 100 mL dioxane/water 3:4 at 5 °C. The mixture was stirred at rt for 15 h and the dioxane was removed in vacuo. The residue was dissolved in CH_2_Cl_2_ and washed with the brine. Removal of solvent and column chromatography using an appropriate mixture of ethyl acetate/petroleum ether gave the pure lactam **12** (0.33 g, 34%) as a semi solid. IR (neat): 3020 (m), 2400 (w), 2125 (w), 1678 (w), 1422 (w), 1216 (m), 1049 (w), 1022 (w), 929 (w), 759 (s) cm^−1^; ^1^H NMR (500 MHz, CDCl_3_) δ 6.36–6.34 (m, 1H), 6.15 (dd, *J* = 5.5, 3 Hz, 1H), 6.07 (dd, *J* = 5.5, 3 Hz, 1H), 5.96 (bs, 1H), 5.63 (dt, *J* = 8.5, 2 Hz, 1H), 4.12–4.08 (m, 1H), 3.10 (t, *J* = 0.5 Hz, 1H), 3.06 (d, *J* = 0.5 Hz, 1H), 2.99–2.95 (m, 1H), 1.44 (dt, *J* = 8.5, 2Hz, 1H), 1.25–1.22 (m, 1H) pmm; ^13^C NMR (125 Hz, CDCl_3_) δ 164.4, 142.4, 136.9, 134.5, 122.4, 54.9, 49.8, 47.8, 44.5, 39.3 ppm; HRMS (Q-Tof) *m*/*z*: [M + Na]^+^ calcd for C_10_H_11_NNaO, 184.0733; found, 184.0733.

### Synthesis of compound **2**

Analogously as described in [8], a suspension of NaH (20 mg, 0.83 mmol) in dry DMF (5mL), was added to compound **12** (70 mg, 0.43 mmol) in dry DMF (5 mL) and allyl bromide (57 mg, 0.47 mmol) at 0 °C under nitrogen and it was stirred for 20 minutes at 0 °C. After completion of the reaction (TLC monitoring) the reaction mixture was acidified with saturated ammonium chloride and extracted with ethyl acetate. The combined organic layer was washed with water and brine and then dried over sodium sulfate. Later, the organic layer was concentrated under reduced pressure and purified by silica gel column chromatography by eluting with an appropriate mixture of ethyl acetate/petroleum ether to afford compound **2** as a brown liquid (87 mg, 80%). IR (neat): 3370 (s), 2945 (m), 2832 (m), 2532 (w), 2044 (w), 1662 (w), 1450 (m), 1114 (m), 1030 (s), 770 (m) cm^−1^; ^1^H NMR (500 MHz, CDCl_3_) δ 6.25–6.23 (m, 1H), 6.05–6.01 (m, 2H), 5.85–5.77 (m, 1H), 5.67 (dd, *J* = 10, 2 Hz, 1H), 5.26–5.22 (m, 2H), 4.47–4.46 (m, 1H), 4.02 (dd, *J* = 10, 3.5 Hz, 1H), 3.65–3.60 (m, 1H), 3.29 (s, 1H), 3.08 (s, 1H), 3.01–2.97 (m, 1H), 1.45 (dt, *J* = 9, 2 Hz, 1H), 1.21–1.24 (m, 1H) ppm; ^13^C NMR (125 Hz, CDCl_3_) δ 162.5, 140.1, 137.1, 133.8, 133.6, 123.1, 117.7, 59.43, 48.4, 47.4, 47.3, 44.7, 40.0 ppm; HRMS (Q-Tof) *m*/*z*: [M + Na]^+^ calcd for C_13_H_15_NNaO, 224.1046; found, 224.1041.

### Synthesis of compound **1**

Analogously as described in [8], to a stirred solution of compound **2** (20 mg, 0.099 mmol) in dry CH_2_Cl_2_ (20 mL) degassed with nitrogen for 10 minutes, purged with ethylene gas for 10 minutes was then added Ti(OiPr)_4_ and Grubbs-II catalyst (8.4 mg, 10 mol %) and stirred for 5 h at reflux conditions under ethylene atmosphere. After completion of the reaction (TLC monitoring) the solvent was removed on a rotavapor under reduced pressure and purified by silica gel column chromatography by eluting with an appropriate mixture of ethyl acetate/petroleum ether to afford **1** as a brown coloured semi solid (18 mg, 90%). IR (neat): 3020 (m), 2927 (m), 2861 (m), 2396 (w), 1727 (w), 1608 (w), 1461 (w), 1216 (m), 929 (w), 762 (s) cm^−1^; ^1^H NMR (500 MHz, CDCl_3_) δ 6.35–6.27 (m, 1H), 6.05–5.89 (m, 1H), 5.88–5.83 (m, 1H), 5.75–5.72 (m, 1H), 5.63 (dt, *J* = 16.0, 9.7 Hz, 1H), 5.02–4.91 (m, 2H), 4.64–4.57 (m, 1H), 4.07–4.03 (m , 1H), 3.50–3.42 (m, 1H), 3.19–3.14 (m, 1H), 3.12–2.94 (m, 1H), 2.62–2.55 (m, 1H), 2.21–2.03 (m, 1H), 1.62–1.53 (m, 1H) ppm; ^13^C NMR (125 Hz, CDCl_3_) δ 164.2, 139.6, 139.6, 125.7, 123.5, 123.2, 115.5, 59.0, 58.8, 49.1, 42.3, 40.9, 39.6 ppm; HRMS (Q-Tof) *m*/*z*: [M + Na]^+^ calcd for C_13_H_15_NNaO, 224.1046; found, 224.1041.

## Supporting Information

File 1 NMR spectra of synthesized compounds and X-ray data of compound **11b**.
