# Supplementary material for: Synthesis of a tricyclic lactam via Beckmann rearrangement and ring-rearrangement metathesis as key steps
Source: Beilstein J Org Chem. 2015 Aug 27;11:1503–8. doi: 10.3762/bjoc.11.163 (PMC4578362; doi:10.3762/bjoc.11.163)
Supplement: File 1 — NMR spectra of synthesized compounds and X-ray data of compound 11b. [file Beilstein_J_Org_Chem-11-1503-s001.pdf]

Supporting Information

for

Synthesis of a tricyclic lactam via Beckmann rearrangement and ring-rearrangement metathesis as key steps

Sambasivarao Kotha*, Ongolu Ravikumar and Jadab Majhi

Address: Department of Chemistry, Indian Institute of Technology-Bombay, Powai, India,

Fax: 022-25767152

Email: Sambasivarao Kotha - srk@chem.iitb.ac.in

*Corresponding author

NMR spectra of synthesized compounds and X-ray data of compound 11b

Compound 9a ^{1}H and ^{13}C NMR

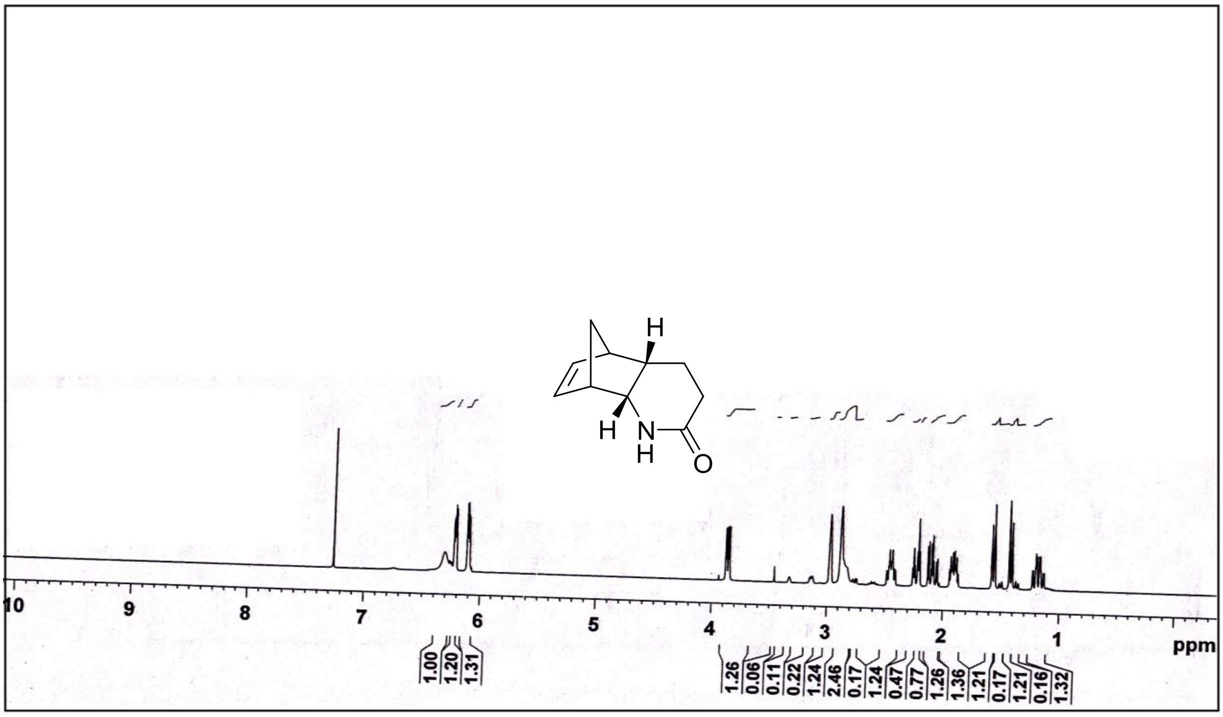

Compound 11a ^{1}H and ^{13}C NMR

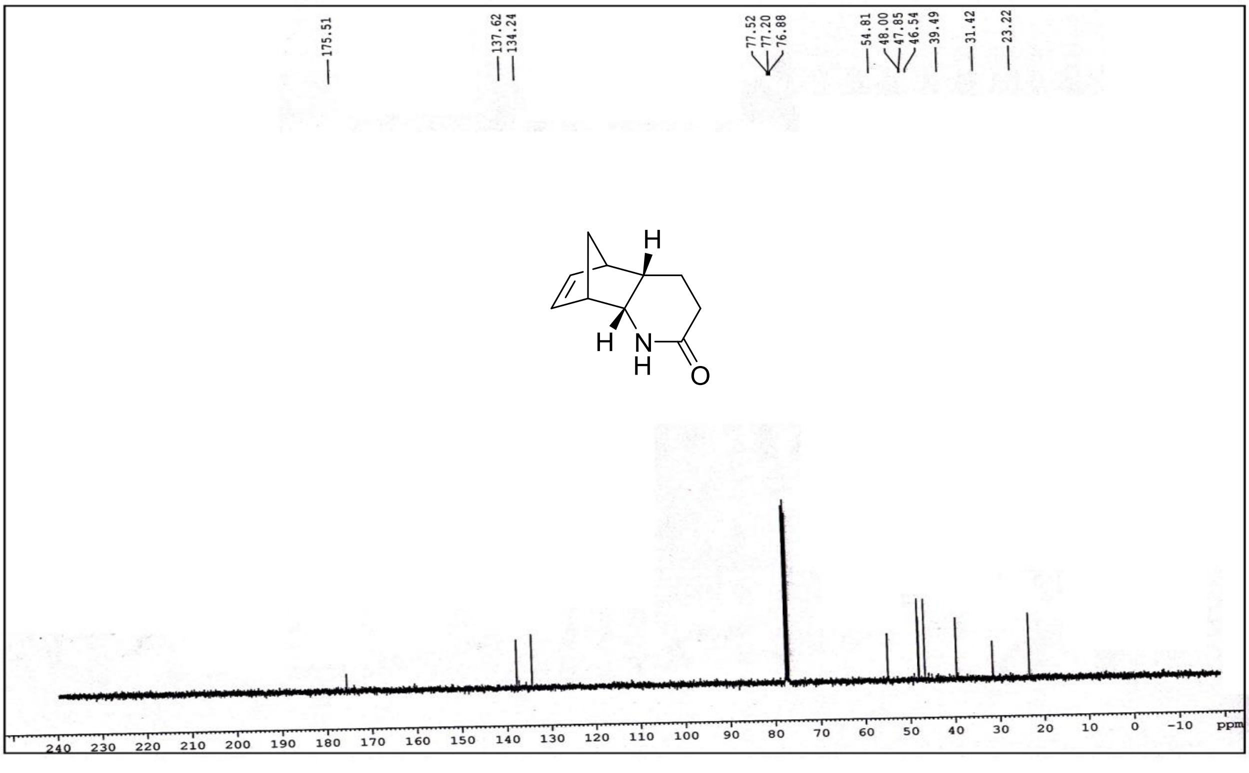

Compound 11a ^{1}H and ^{13}C NMR

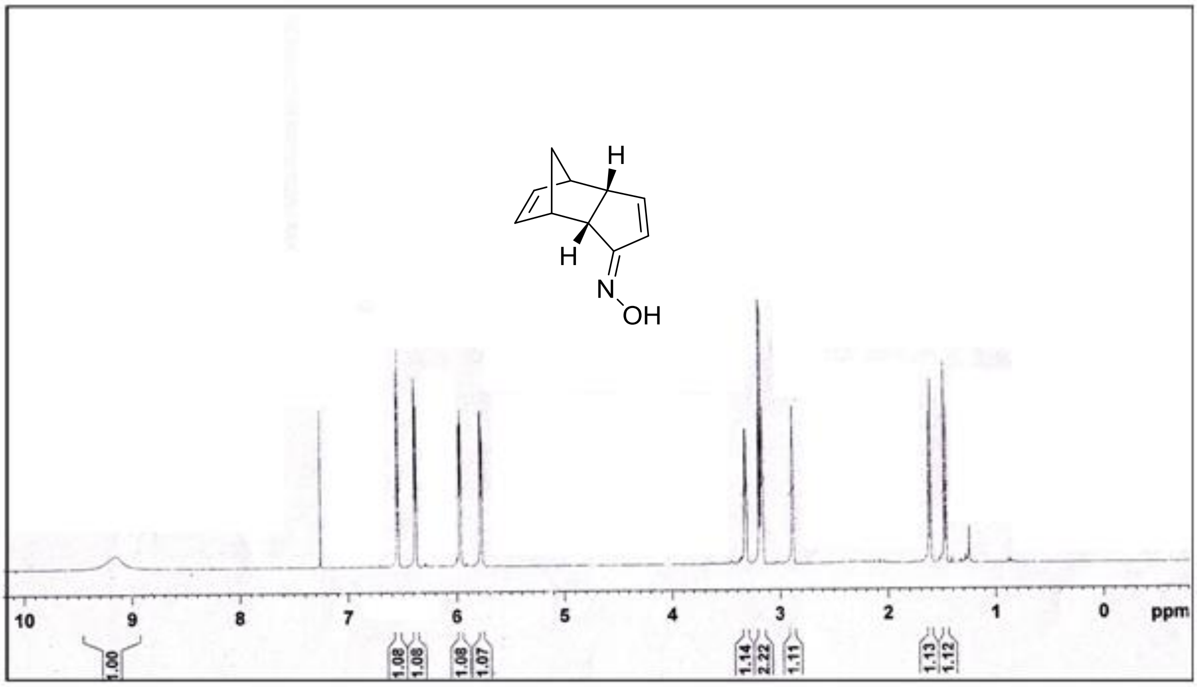

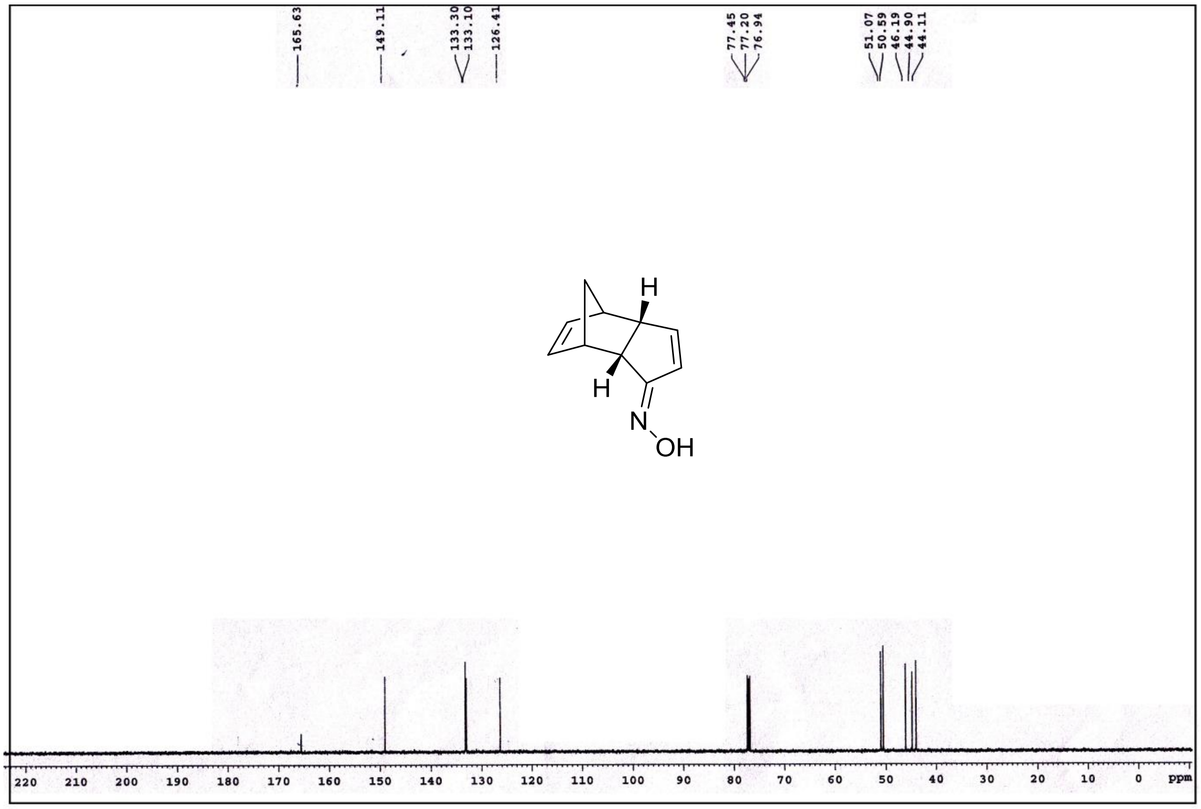

Compound 11b ^{1}H and ^{13}C NMR

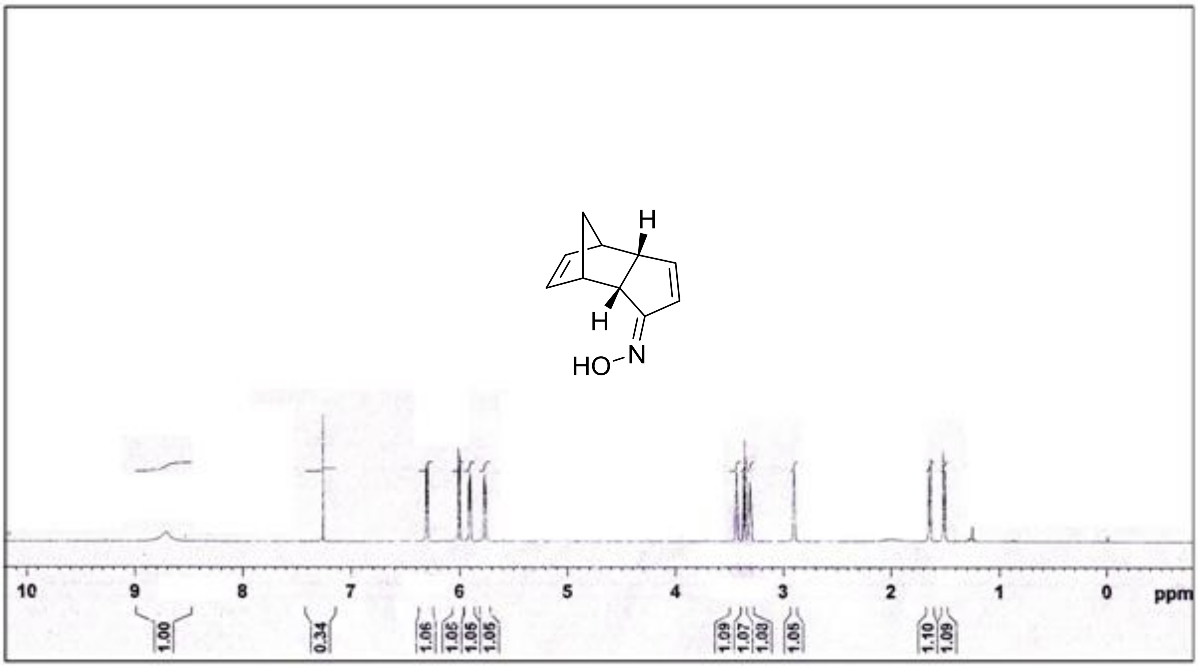

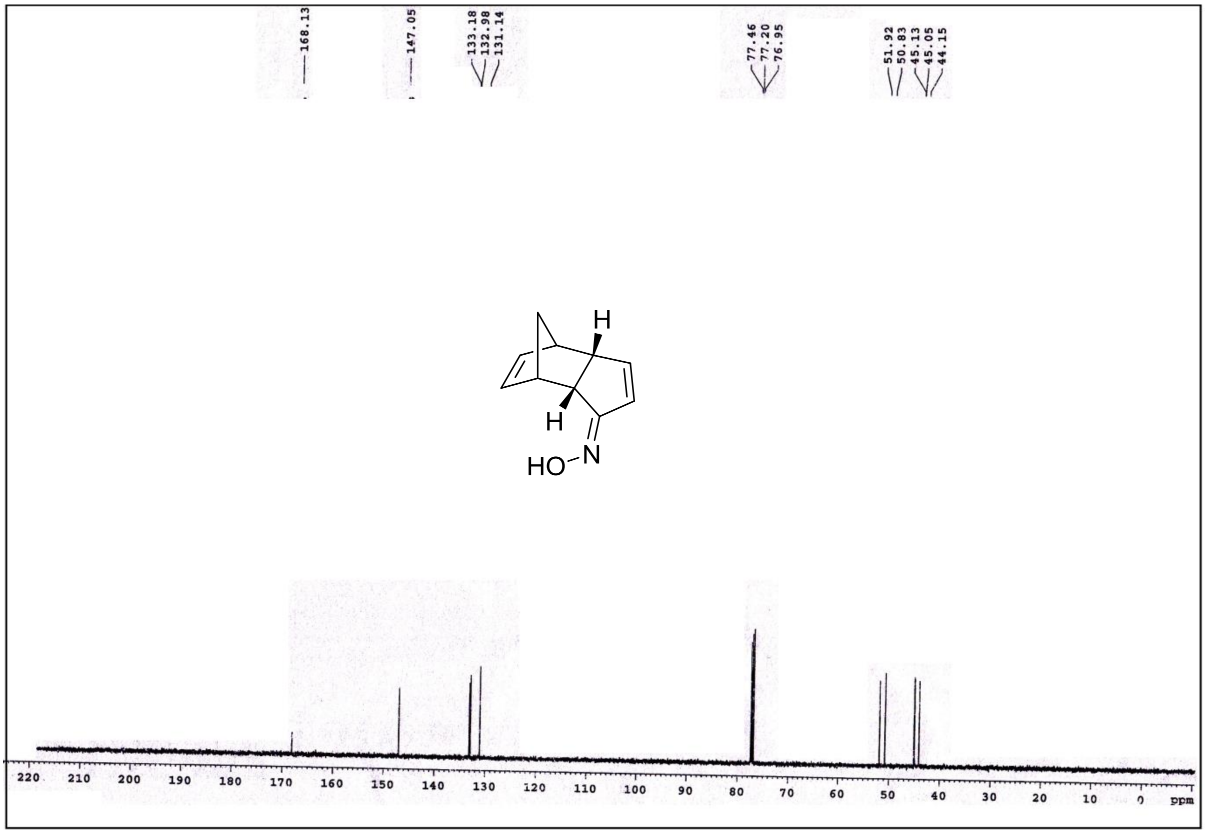

Compound 12 ^{1}H and ^{13}C NMR

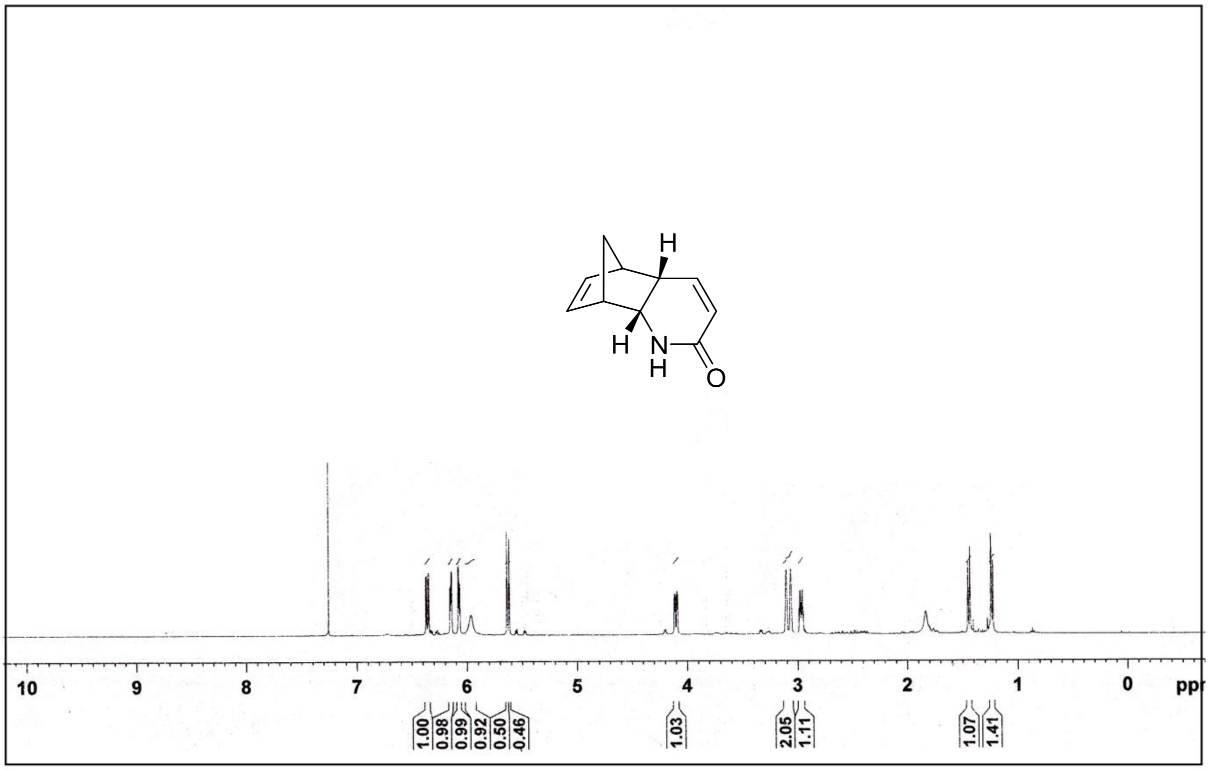

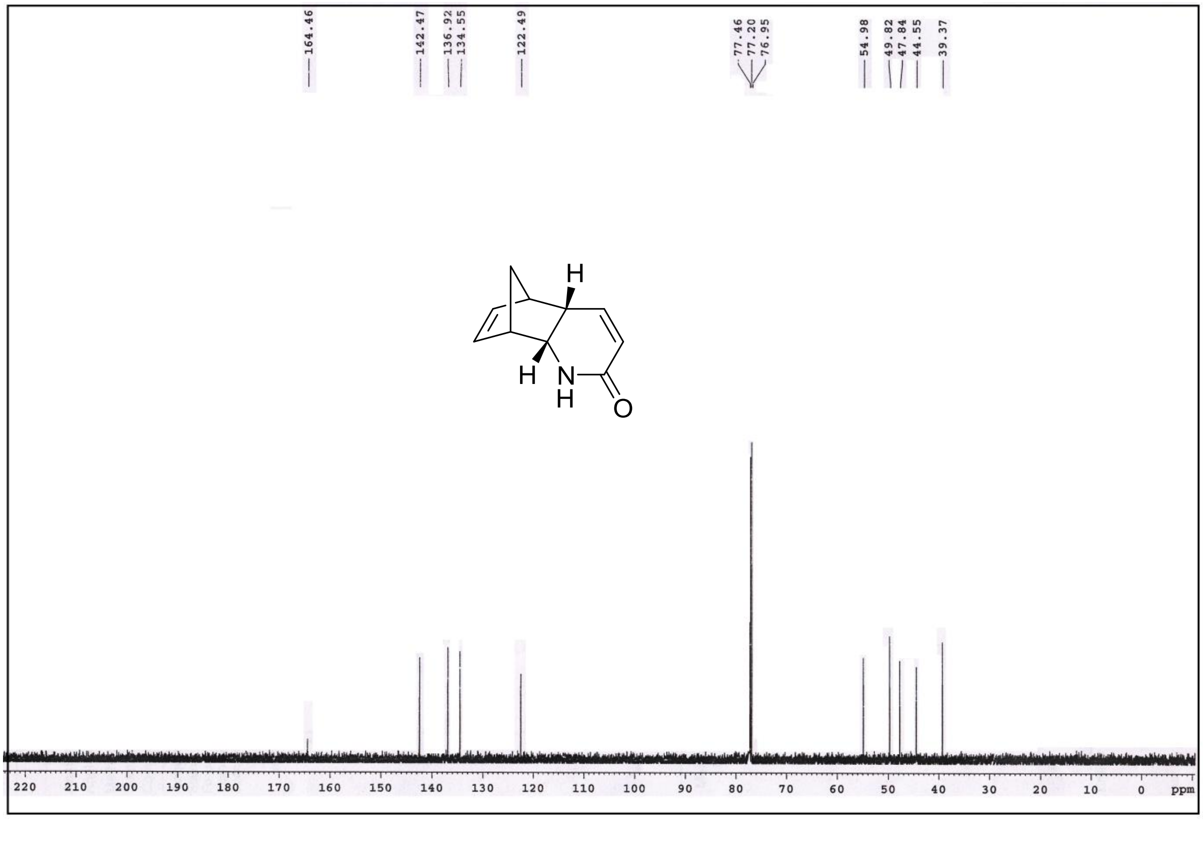

Compound 2 ^{1}H and ^{13}C NMR

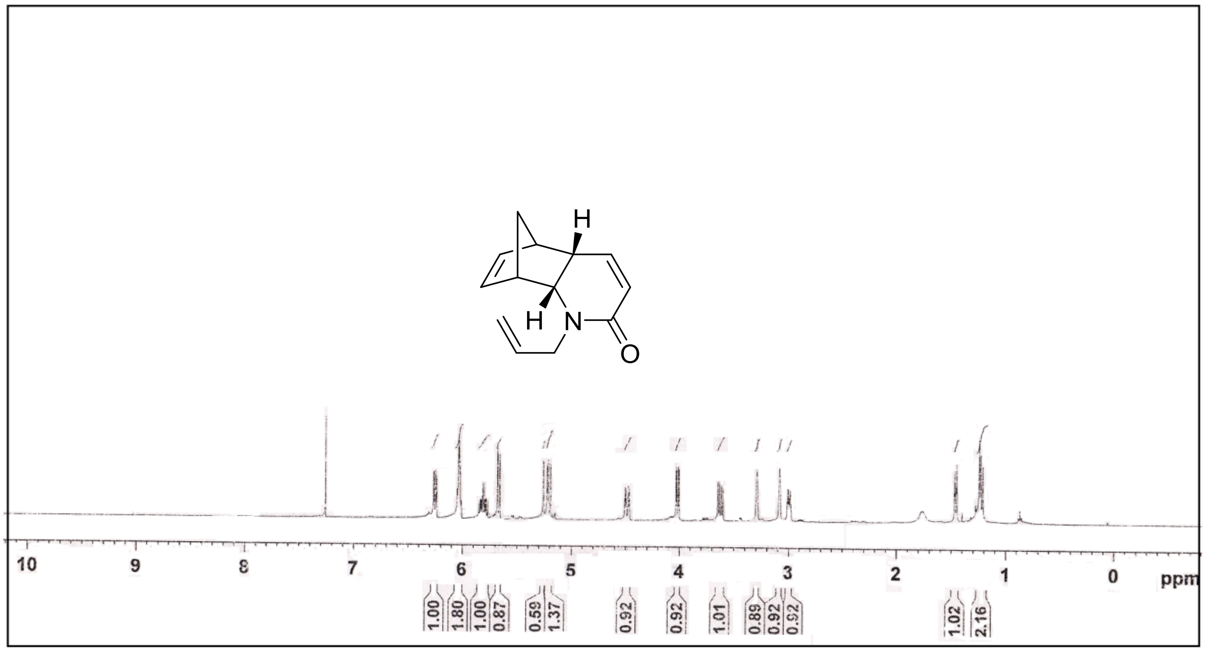

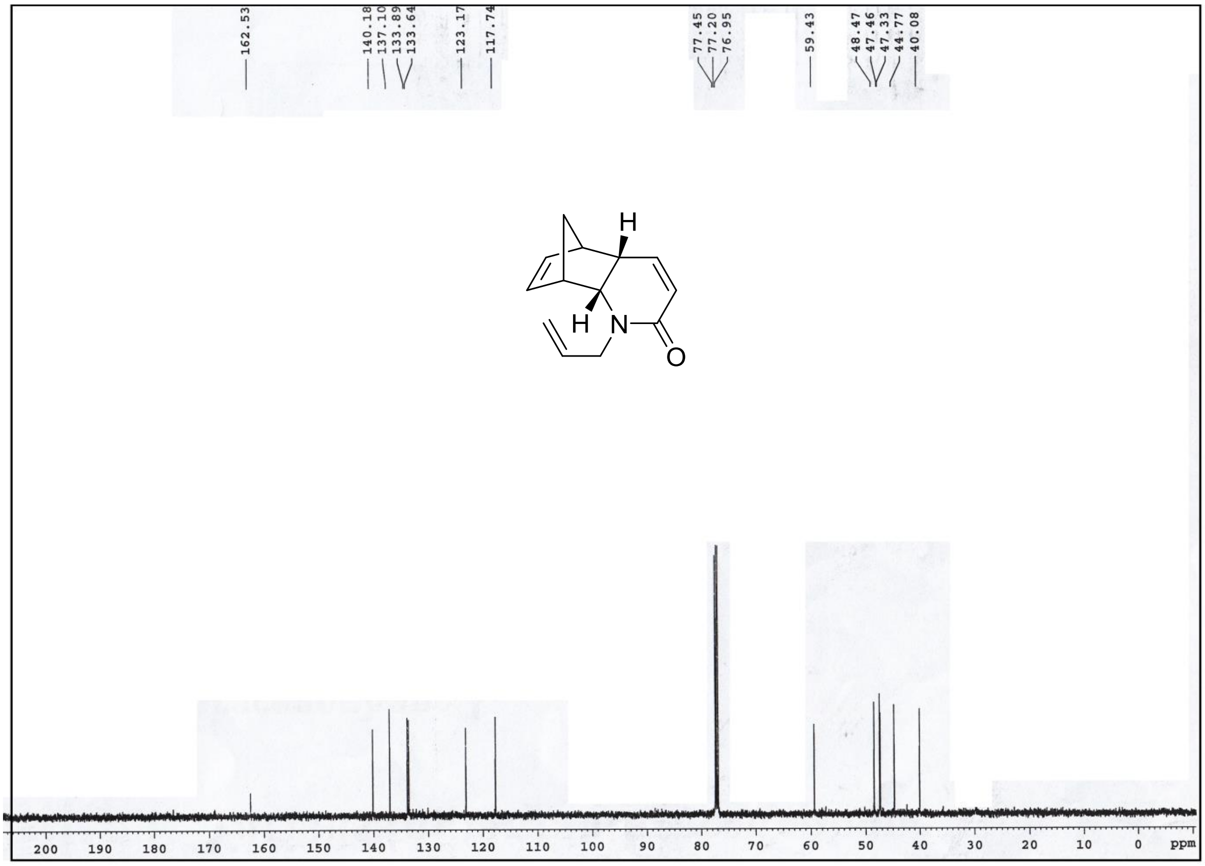

Compound 1 ^{1}H and ^{13}C NMR

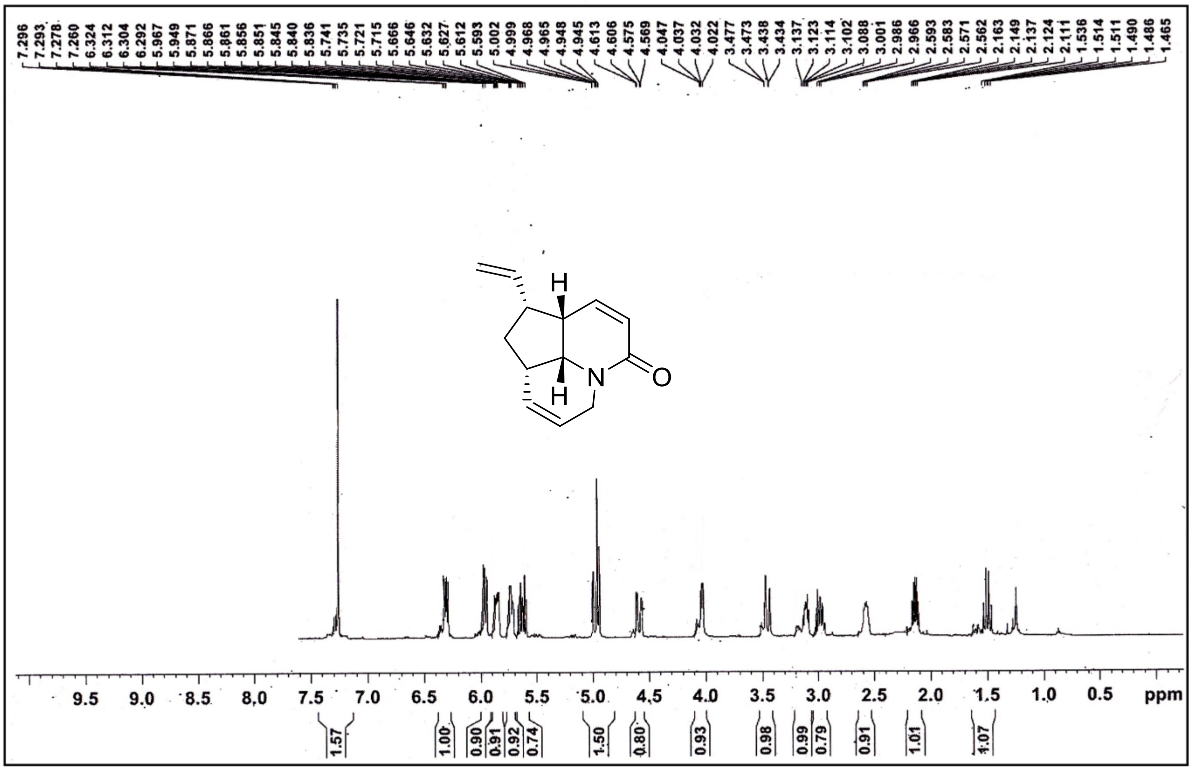

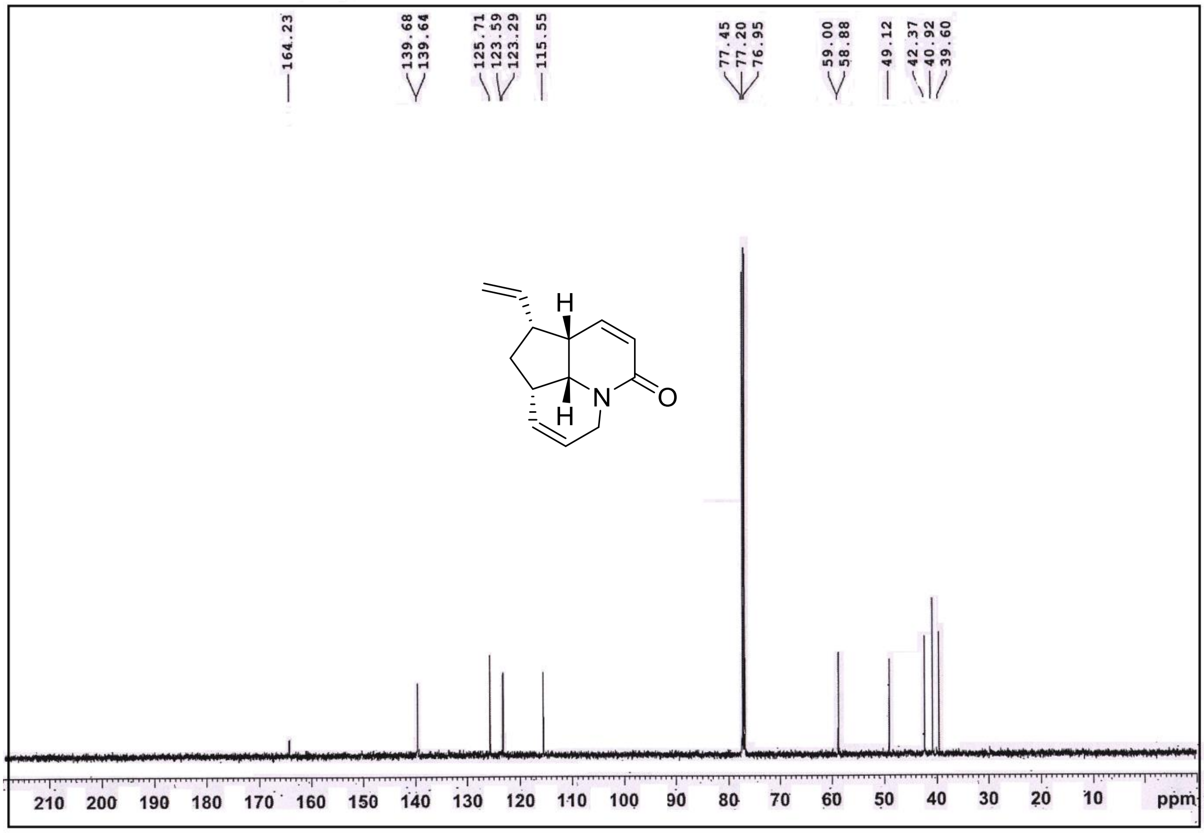

X-ray crystallographic data and refinement parameters for compound 11b (CCDC 1403298)

Bond precision:	C-C = 0.0051 A		Wavelength=1.54190
Cell:	a = 6.081(2)	b = 21.357(11)	c = 19.131(8)
	α = 90	β = 92.969(7)	ɤ = 90
Temperature:	100 K		

	**Calculated**	**Reported**
Volume	2481.2 (18)	2481.2 (19)
Space group	P 21/c	P 1 21/c 1
Hall group	-P 2ybc	-P 2ybc
Moiety formula	$C_{10} H_{11} NO$	$C_{10} H_{11} NO$
Sum formula	$C_{10} H_{11} NO$	$C_{10} H_{11} NO$
Mr	161.20	161.20
Dx,g cm^{-3}	1.295	1. 294
Z	12	12
Mu (mm-1)	0.669	0.670
F000	1032.0	1032.0
F000’	1034.96	
h, k, lmax	7, 25, 23	7, 25, 23
Nref	4573	4514
Tmin, Tmax	0.892, 0.898	0.858, 0.898
Tmin’	0.892	

Correction method = Not given

Data completeness = 0.987 Theta (max) = 68.570

R(reflections) = 0.0798(3563) wR2(reflections) = 0.2954(4514)

S = 2.10 Npar = 325
